# Improved cognition after rifaximin treatment is associated with changes in intra- and inter-brain network functional connectivity

**DOI:** 10.1186/s12967-023-04844-7

**Published:** 2024-01-12

**Authors:** Franc Casanova-Ferrer, Juan-José Gallego, Alessandra Fiorillo, Amparo Urios, María-Pilar Ríos, José Luis León, María-Pilar Ballester, Desamparados Escudero-García, Elena Kosenko, Vicente Belloch, Carmina Montoliu

**Affiliations:** 1 Fundacion de Investigación Hospital Clinico Universitario de Valencia-INCLIVA, Valencia, Spain; 2 Servicio de Medicina Digestiva, Hospital Arnau de Vilanova de Valencia, Valencia, Spain; 3 Universitats Neurorradiology Unit, Ascires Biomedical Group, Valencia, Spain; 4 Servicio de Medicina Digestiva, Hospital Clinico Universitario de Valencia, Valencia, Spain; 5 Departamento de Medicina, University of Valencia, Valencia, Spain; 6 Institute of Theoretical and Experimental Biophysics of Russian Academy of Sciences, Pushchino, Russia; 7 Department of Pathology, Faculty of Medicine, University of Valencia, Av Blasco Ibáñez, 15, 46010 Valencia, Spain

**Keywords:** Rifaximin, Psychometric tests, Functional magnetic resonance imaging, Minimal hepatic encephalopathy

## Abstract

**Background:**

Rifaximin is a non-reabsorbable antibiotic which acts at gut level, and improves cognition and inflammatory parameters in minimal hepatic encephalopathy (MHE) patients, but not all patients show the same level of response. This study aims to assess brain activity, both within and between brain networks, following rifaximin treatment, considering the differences between response groups as well.

**Methods:**

Twenty-two healthy controls and 53 patients with cirrhosis (22 without and 31 with MHE, diagnosed by Psychometric Hepatic Encephalopathy Score, PHES) performed psychometric, attention and coordination tests, and blood inflammatory parameters were measured. Resting-state functional magnetic resonance imaging (fMRI) acquisitions were performed on controls and MHE patients. Eighteen MHE patients underwent a rifaximin treatment for 6 months, after which all measures were repeated. fMRI images were analysed and changes after treatment were assessed.

**Results:**

After rifaximin treatment, 13 patients improved their PHES score (Responder patients) while 5 did not (Non-responder patients). No significant decrease in blood ammonia was observed after rifaximin treatment, but there was a decrease in plasma inflammatory cytokines in responder patients. A global effect of rifaximin was detected on the sensorimotor and fronto-parietal networks. Responder patients showed a relative increase of thalamic network connectivity in comparison to non-responder patients. Before treatment, responder and non-responder patients showed connectivity differences in basal ganglia network. The connection of the sensorimotor and thalamic networks between them and with other networks suffered changes after treatment. These connections between networks mostly decreased after treatment. All changes and differences showed a significant level of correlation with the performance of psychometric tests and the blood levels of inflammatory biomarkers.

**Conclusions:**

There was an improvement of the communication between executive, motor and attention-related brain areas, and their functional independence following rifaximin treatment. Patients who respond also show a less deteriorated connection involved in these functions before treatment. Results suggest that the improved inflammatory state of MHE patients, following rifaximin treatment would favour the observed changes in brain function and enhanced cognitive performance.

**Supplementary Information:**

The online version contains supplementary material available at 10.1186/s12967-023-04844-7.

## Introduction

Between a third and half of patients with liver cirrhosis develop minimal hepatic encephalopathy (MHE), which is defined as the preclinical state of hepatic encephalopathy (HE) [1–3], and is characterized by mild cognitive impairment including alterations in attention, motor performance and balance [4–7]. These alterations are associated with a higher risk of falls, impaired driving ability, and a general deterioration in quality of life of patients [8–11]. Moreover, patients with MHE have an increased risk of developing HE [12]. Early detection of MHE and treatment can help reduce hospitalization costs, prolong life expectancy in patients, and improve overall quality of life [13, 14].

Advances in functional magnetic resonance imaging (fMRI) technology over recent years has made it possible to study functional changes in the brain and cerebellum of people with pathologies affecting neurological function such as MHE, and associate these changes with cognitive alterations specific to each patient. Of particular interest is the emergence of resting-state fMRI, a technique for studying the function of various brain networks and the connections between them without the need for patients to perform any tasks during analysis. Recent studies in MHE have described structural changes such as volume reduction in the hippocampus, focal damage in the precuneus, and microstructural alterations in white matter [15–17]. Brain networks such as the default mode network, the attention network, the visual network, the hippocampus and the thalamus have also shown altered function in MHE patients [18–23]. Some of these alterations correlate with cognitive impairment and memory performance and also with plasma levels of pro-inflammatory cytokines [15, 22]. These studies were performed in patients with liver cirrhosis due to several aetiologies, mainly alcohol-, HCV- and HBV-related cirrhosis [18–23]. Ahluwalia et al. [24] found that brain reserve as shown using the MRI neurometabolic and neurostructural profile is significantly impaired in abstinent alcoholic patients with cirrhosis compared to nonalcoholic patients with cirrhosis. MRI results in that study showed a greater effect of hyperammonemia, brain edema, and significantly higher cortical damage in alcoholic-related cirrhosis as compared to nonalcoholic patients. Studies in patients with HBV-related cirrhosis show abnormalities in subcortical and cortical functional networks, which correlate with disease duration and psychometric tests [25]. HCV-related cognitive decline is associated with neuroinflammation and structural disintegrity in basal ganglia, frontal and occipital white matter [26], but functional MRI studies in HCV patients with MHE are scant.

The underlying mechanisms of alterations in neuronal connectivity associated to MHE are not known. Alterations in the gut-liver-brain axis seem to play a relevant role in the induction of MHE [27]. Gut microbiome is altered in patients with liver cirrhosis which may contribute to alterations in the immune system and cognition [27]. Peripheral inflammation and hyperammonemia play synergistic roles in inducing MHE [28, 29], and we previously showed that MHE appearance is associated with specific changes in immune system and peripheral inflammation [30]. In animal models of hyperammonemia and MHE, it was shown that peripheral alterations are transmitted to brain inducing neuroinflammation, which alters neurotransmission, leading to cognitive and motor impairment [31]. A similar process would occur in MHE patients. Patients died with liver disease show neuroinflammation in cerebellum, with activation of microglia and astrocytes and loss of Purkinje and granular neurons [32]. Based on these studies, the sequence of events that would induce MHE could be the following: alterations in immunological system and in inflammatory parameters associated with MHE would be transmitted to brain, leading to alterations in neurotransmission and functional connectivity, which would trigger cognitive and motor alterations.

Together with the early detection provided by the Psychometric Hepatic Encephalopathy Score (PHES) as the “gold standard” for the diagnosis of MHE [33, 34], some treatments have shown an ability to prevent MHE progression towards HE. According to clinical practice guidelines [1], treatment can be initiated with lactulose and/or rifaximin on suspicion of MHE. Both treatments exert their effects mainly by regulating the activity of the gut microbiota, and have proven effective for reversal of MHE [35–37]. No remarkable differences in the effectivity of one over the other can be found in the literature [38], although rifaximin is better tolerated than lactulose [39].

Some clinical trials studied the effect of rifaximin in patients with MHE. They reported an improvement in driving and cognitive skills, quality of life and reduction of endotoxins [37, 40]. Other studies showed that rifaximin prevented HE episodes and relapses [36, 41]. We previously showed that rifaximin treatment reverses immunophenotype and inflammatory alterations and improves cognitive function in some MHE patients but not in others [42], and that patients with clinical signs of metabolic syndrome have a poor response to rifaximin for MHE [43]. Moreover, after rifaximin treatment, there was a decrease in a parameter of axonal injury in patients who responded to treatment [44].

The present study investigates the effects of rifaximin treatment on brain function in MHE patients using fMRI techniques. We analysed whether improvement by rifaximin of cognitive function and peripheral inflammation is associated with changes in brain functional connectivity. We analysed both independent brain network function and the functional connections between these networks, the latter representing a new approach to study of the effects of MHE and rifaximin. We also examined both pre- and post-treatment differences between patients who respond favourably or not to rifaximin treatment. Finally, we ascertained the correlation between these changes and improvements in psychometric performance and inflammatory parameters after treatment.

## Patients and methods

### Participants

**Fig. 1:**
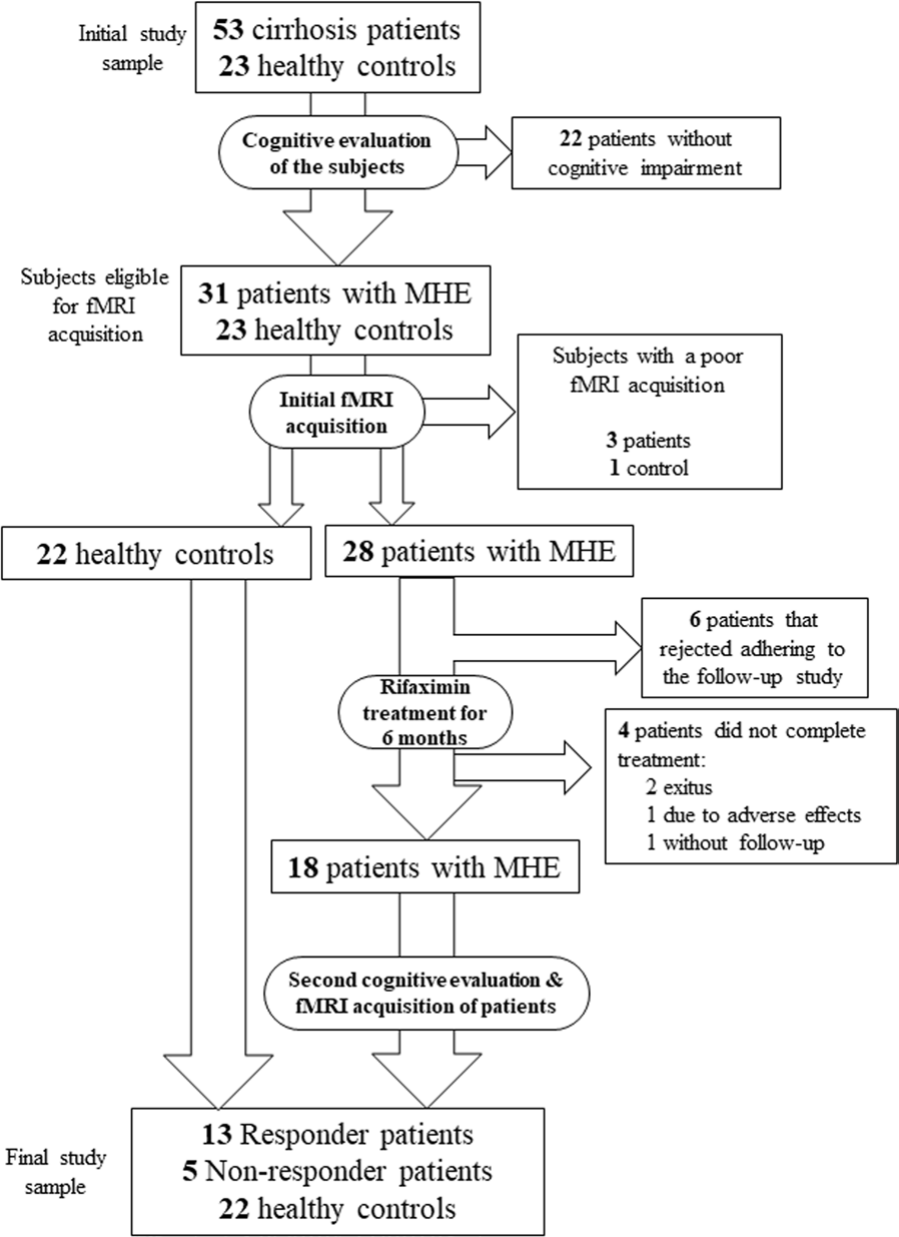
Flow chart of the steps followed in the selection of the study sample and follow-up that led to the final study groups

A total of 53 patients with liver cirrhosis and 23 healthy controls without liver disease were enrolled as volunteers onto the study after written informed consent. Patients were recruited between July 2015 and January 2019 from the outpatient clinics of Hospital Clinico Universitario and Hospital Arnau de Vilanova, in Valencia, Spain. Inclusion criteria were clinical, biochemical, and histological evidence of liver cirrhosis. For healthy controls, liver disease was discarded via clinical, analytical, and serologic analysis. Exclusion criteria included HE or history of HE, alcohol intake during the 6 months prior to recruitment, infections, antibiotic use or gastrointestinal bleeding during the 6 weeks prior to recruitment, history of shunt surgery or transjugular intrahepatic portosystemic shunt for portal hypertension, use of drugs that affect cognitive function, hepatocellular carcinoma, and neurological or psychiatric disorders. Patients included in the study before and after rifaximin treatment, did not show fever or any clinical or biological sign of recent infection. Psychometric, attention and coordination tests, and blood collection were performed on the same day. Twenty-two patients were classified as without MHE (nMHE) and 31 as with MHE using the PHES battery (see below) [33, 34]. fMRI acquisition was performed on healthy controls and MHE patients in the week following neuropsychological assessment. After fMRI acquisition, four subjects (one healthy control and three MHE patients) were excluded from the study due to poor acquisition caused by excessive head movement during the process (translation > 2.5 mm or rotation > 2.5˚). After this reduction, 22 healthy controls and 28 patients remained (Fig. 1).

Study protocols were approved by Scientific and Research Ethics Committees of Hospitals Clinico and Arnau Vilanova, Valencia, Spain, (F-CE-GEva-15; 2018.51) and classified by the Spanish Agency of Medicines and Medical Devices (CMF-NRT-2017). The study protocol conforms to the ethical guidelines of the 1975 Declaration of Helsinki. The demographic characteristics and disease aetiology of each group are shown in Table 1.

**Table 1:** Demographic characteristics and liver disease aetiology by group

	Controls (n = 22)	nMHE patients (n = 22)	MHE patients (n = 31)	MHE patients following treatment (n = 18)	
				Response (n = 13)	No response (n = 5)
Sex (M/F)	14/8	15/7	26/5	11/2	5/0
Age^†^	60 ± 6 (50–73)	62 ± 8 (50–81)	64 ± 9 (48–85)	62 ± 7 (53–74)	65 ± 10 (49–74)
Aetiology					
Alcohol		7	13	8	1
Hepatitis (HCV/HBV)		11/0	9/1	4/0	0/1
Metabolic		2	6	0	2
Other		2	2	1	1
Child Pugh A/B/C^‡^		19/3/0	17/10/4*	8/5/0	4/1/0
MELD^†^^,^^§^		8 ± 2	10 ± 4*	9 ± 3	8 ± 2

In brackets: age range

Comparisons between controls, nMHE, and MHE groups were analysed by one-way ANOVA followed by post-hoc Tukey’s multiple comparison test

HBV, hepatitis B virus; HCV, hepatitis C virus; MHE, minimal hepatic encephalopathy; MELD, model end stage liver disease

The Child Pugh Score is derived from a score of 1–3 given for severity of ascites, hepatic encephalopathy, INR, albumin and bilirubin. The higher the score, the greater the liver disease severity

Significant differences are indicated by *: *p < 0.05

^†^Values are expressed as mean ± SD

^‡^Differences in proportions were analysed with Chi-square test

^§^Differences between groups (nMHE vs. MHE; response vs. no response groups) were analysed with *T*-test

### Diagnosis of MHE

MHE was diagnosed using the PHES battery of tests [33, 34]. Scores were adjusted for age and education level using Spanish normality tables (https://www.redeh.org/TEST_phes.htm. Accessed on 14 July 2023). Patients were classified as MHE when PHES score was ≤  − 4 points.

Additional psychometric tests performed were focused on different cognitive functions: cognitive flexibility and inhibitory control (Stroop test); selective, sustained attention and mental concentration (d2 test); mental processing speed (Oral Symbol Modalities test, SDMT); working memory (digit span and letter-number sequencing test, from Wechsler Adults Intelligence Scale), and bimanual and visuomotor coordination tests. All tests were performed as previously described [45].

### Ammonia and pro-inflammatory cytokines level measurement

Blood ammonia levels were measured immediately after blood collection using the Ammonia Test Kit II for the PocketChemBA system (Arkay, Inc., Kyoto, Japan). Plasma concentrations of IL-6, IL-18, IL-22 (Affymetrix eBioscience, Vienna, Austria), IL-15, CCL20, CXCL13 and CX3CL1 (R&D Systems, Minneapolis, MN, USA) were measured by ELISA according to the manufacturer’s instructions.

### Rifaximin treatment

Of the 28 MHE patients, 22 were prescribed rifaximin treatment (1.2 g/day, in three doses of 400 mg every 8 h) after the first fMRI acquisition session. The remaining 6 subjects underwent the prior acquisition of MRI, but they subsequently refused to continue in the study, so these patients could not be followed up, regardless of whether or not they had been prescribed treatment. After 6 months of treatment, patients underwent a second psychometric evaluation and fMRI acquisition session, during which four patients dropped out of the study: one due to adverse effects, two died and one declined to undergo a second interview and acquisition session. This reduced the study group to a total of 18 patients with known treatment response and follow-up fMRI acquisition, of which 13 responded favourably to rifaximin (responders), while the other five showed a lack of response (non-responders). Patients whose PHES score results were consistent with nMHE patients (PHES score > − 4) were classified as responders, while those not matching these criteria were classified as non-responders (Fig. 1). The demographic characteristics and disease aetiology of response groups are in Table 1.

No HE episodes occurred during the 6 months of treatment with rifaximin, and there were few cases of other decompensations, such as ascites (2 patients) or portal thrombosis (1 patient), all occurring in the responder group, which indicates that most patients were clinically stable.

### Image acquisition

All subjects underwent an MRI scan using a 3 T Philips Achieva scanner (Philips Medical Systems, Netherlands). Sagittal high-resolution three-dimensional 3D MPRAGE T1 images were acquired (TR = 8.42 ms, TE = 3.8 ms, matrix = 320 × 320 × 250, voxel size = 1 × 1 × 1 mm, flip angle = 8˚). In addition, functional MRI resting-state data was acquired using a gradient-echo T2-weighted echo-planar imaging (EPI) sequence (5 min, 150 volumes, TR = 2000 ms, TE = 30 ms, matrix = 80 × 80 × 31, voxel size = 3 × 3 × 3 mm, flip angle = 85˚). During the resting sequence, participants were instructed to remain motionless and relax with their eyes open, not fall asleep and think of nothing in particular.

### Image pre-processing

All processing and data analysis of fMRI data were conducted using the Oxford Centre for Functional MRI of the Brain (FMRIB) Software Library (FSL) version 6.0.1, and third party tools specially developed for this software [46].

The first ten images of the fMRI time series were discarded to account for magnetic saturation effects. Remaining volumes were motion-corrected using MCFLIRT [47]. Brain extraction, or cropping, was then performed on motion-corrected fMRI volumes and structural images using FMRIB’s Brain Extraction Tool [48] and interleaved slice timing correction was conducted. Volumes were spatially smoothed with a 4 mm full width at half maximum Gaussian kernel, and high-pass filtered with a cut-off of 100 s. For their registration to standard space, functional images were linearly registered to their corresponding structural images. Afterwards, non-linear registration of the structural images to MNI152 standard space was performed. Finally, functional images were non-linearly registered to MNI152 standard space using the previous registration of their corresponding structural images. All steps described were applied as part of the FSL Multivariate Exploratory Linear Optimized Decomposition into Independent Components (MELODIC) tool process, which applied single-subject independent component analysis (ICA) on the images resulting from the previously described steps. Automatic estimation of dimensionality was used in this analysis.

The resulting images from MELODIC were denoised using ICA-AROMA [49], which performs another single-subject ICA to remove motion-related components.

### Analysis of fMRI data

**Fig. 2:**
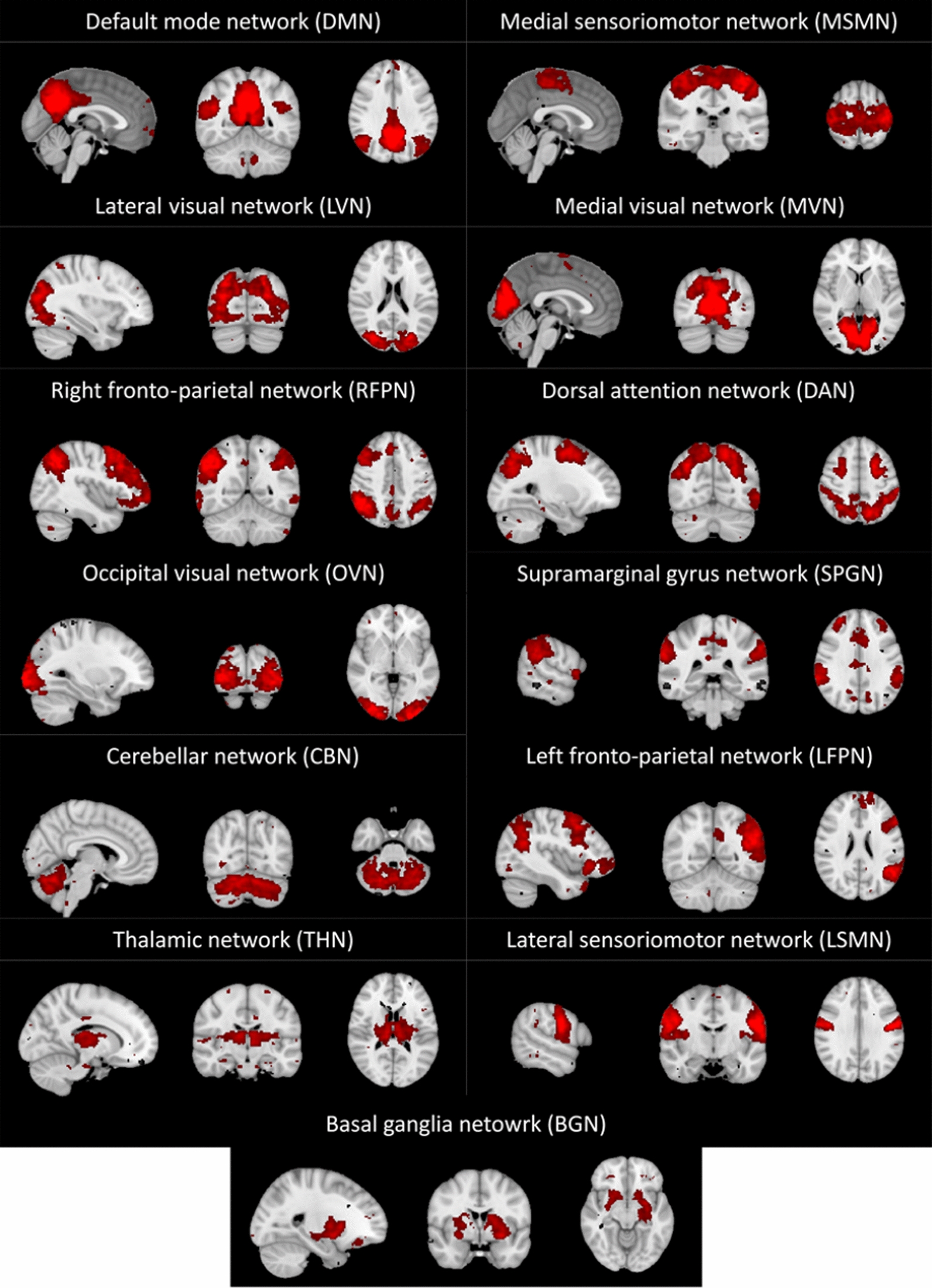
Spatial maps of the 13 resting-state networks identified and analysed in the study. Maps are thresholded at 3 < Z < 10. Images are shown following MNI convention

Group-level ICA was applied using MELODIC. Connectivity networks were obtained from the images of a random selection of subjects in which the main study groups were equally represented, as per standard protocol in this methodology (10 healthy controls, 5 responder patients and the 5 non-responder patients). All images used were temporally concatenated, and then split into 20–80 independent components, close to the optimal recommended value for studying connectivity alterations in neuropsychiatric diseases [50]. The model with 60 components yielded the most satisfactory results in terms of quantity and quality of detected resting-state networks (RSNs) upon visual inspection. Thirteen components were considered of biological interest (Fig. 2).

Each of the 13 networks of interest was assigned a name according to its spatial distribution and how it overlapped with RSNs found in reference studies [51, 52]. In cases in which a spatial parallel with these references was not clear, a name was provided depending on the area were the signal of the network was most intense [53].

We next performed FSL dual regression [54]. The spatial maps of all components of biological interest were used as spatial regressors on each subject’s fully pre-processed functional images to obtain the time series and spatial map of each identified RSN of each subject. Time series were obtained using dual-threshold regression for their future use as part of the inter-network functional connectivity analysis. Resulting spatial representations were used for the intra-network functional connectivity analysis.

Intra-network functional connectivity was analysed using FSL Randomise [55]. We applied 5000 permutations, family-wise error correction for multiple comparisons and threshold-free cluster enhancement (TFCE) in every test. The analyses were restricted to voxels present in all subjects included in each specific comparison, using a binary mask previously generated by the program.

Inter-network functional connectivity was analysed using graph theory and the FSLNets package available in MATLAB (https://fsl.fmrib.ox.ac.uk/fsl/fslwiki/FSLNets). The normalised time series obtained during dual-threshold regression were used for this analysis. Partial correlations between each network pair in each subject were calculated and transformed to Fisher’s z-scores for further analyses. We applied 5000 permutations and family-wise error correction for multiple comparisons in all performed tests.

Three main comparisons were performed both when analysing Intra and Inter-network functional connectivity. All comparisons were modelled using general linear models (GLM) created with the GLM function included in FSL. The aforementioned comparisons included:

1. An analysis of the general effect of rifaximin by comparing all patients before and after treatment. In this case we used a GLM similar to a paired t test. The time point of each sample (before vs after treatment) and the identity of each patient, as a correction for the repeated measures analysis, were the factors considered in this case.
2. An analysis of the differences in the effects of rifaximin between responding and non-responding patients. In this case we used a GLM similar to a 2-way mixed effect ANOVA. The time point of each sample (before vs after treatment), the response group of each patient (responding vs non-responding), and the identity of each patient, as a correction for the repeated measures analysis, were the factors considered in this case.
3. An analysis of the pre-existing differences before treatment between responders and non-responders. In this case we used a GLM similar to a t test. The only factor considered in this case was the response group of each patient (responding vs non-responding).

For resting-state networks with significant results in either of the first two analyses during the intra-network functional connectivity analysis, a posterior comparison of post-treatment patients and healthy controls was performed using a GLM similar to a t-test. This final analysis was performed to observe if the changes experimented after treatment were sufficient to overcome alterations previously observed by Garcia-Garcia et al. [15]. In this case, the study group of the subjects (patients *vs*. controls) was the only factor we considered.

In analyses 1 and 2 the images of all patients before and after treatment were used. In analysis 3 only the images obtained before treatment were included. For the posterior comparison of patients and controls we used the images of controls and the images of either all the patients after treatment (posterior comparisons related to analysis 1) or responding patients after treatment (posterior comparisons related to analysis 2). In all cases results were considered significant at p < 0.05 after applying the aforementioned corrections.

Statistically significant clusters were associated with functional regions of the brain cortex or cerebellum using Glasser’s functional parcellation atlas [56], Yeo’s cerebellar atlas [57], and chapters 2–9 of A Connectomic Atlas of the Human Cerebrum [53].

### Correlation analysis

Correlation analyses were performed using the values obtained in fMRI data analysis. The analyses were limited to intra and inter-network connections experiencing significant changes during treatment or that showed significant between- response group differences before treatment, psychometric tests and biochemical determinations. Spearman’s correlation test was performed using R software (version 4.1.1). False discovery rate (FDR) correction was applied on the resulting correlations, and two-sided p values < 0.05 after correction were considered significant.

## Results

### Effects of rifaximin treatment on cognitive tests and inflammatory parameters

MHE patients performed worse in almost all psychometric tests and showed altered levels of all biochemical measurements compared to healthy controls and nMHE patients (Additional file 1: Table S1). A significant increase in PHES score was observed after treatment (p < 0.01), together with better performance of Oral SDMT (p < 0.001) and Stroop test neutral and incongruent tasks (p < 0.05). d2 and Digit Span and letter-number sequencing tests were not affected (Table 2). An improvement in all biochemical measurements (except for ammonia and CXCL13 levels) was observed after treatment (Table 2).

**Table 2:** Psychometric and biochemical characteristics of responding and non-responding MHE patients before and after rifaximin treatment

	General		Responder		Non-responder	
	Before treatment	After treatment	Before treatment	After treatment	Before treatment	After treatment
PHES global score^‡^	− 7.3 ± 0.85	− 4.6 ± 0.76**	− 6.7 ± 0.94	− 3.2 ± 0.66**	− 8.8 ± 1.8	− 8.4 ± 0.68
DST (items completed)^†^	21 ± 2	27 ± 1.9*	22 ± 2.5	30 ± 2*	16 ± 2.8	19 ± 1.9
NCT-A (seconds)^‡^	74 ± 11	57 ± 7*	67 ± 12	50 ± 8.2	93 ± 26	73 ± 11
NCT-B (seconds)^‡^	228 ± 39	159 ± 23	212 ± 44	127 ± 21*	271 ± 85	238 ± 49
SD (seconds)^‡^	119 ± 8.2	107 ± 9.3*	116 ± 11	92 ± 6.5*	127 ± 12	147 ± 21
LTT (seconds + errors)^‡^	210 ± 14	178 ± 16	196 ± 13	155 ± 13*	247 ± 37	237 ± 35
Stroop-congruent task^‡^	76 ± 4.2	82 ± 4.2	80 ± 4.9	86 ± 4.8	67 ± 6.9	69 ± 5
Stroop-neutral task^‡^	56 ± 2.3	62 ± 2.6*	58 ± 2.4	64 ± 3.1*	50 ± 4.7	56 ± 2.8
Stroop-incongruent task^†^	28 ± 2.1	35 ± 2.2*	29 ± 2.1	35 ± 2.1*	24 ± 5	37 ± 7.1
Bimanual coordination (min)^‡^	3.3 ± 0.37	3 ± 0.2	2.8 ± 0.18	2.7 ± 0.13	4.5 ± 1.1^α^	3.8 ± 0.49
Visuo-motor coordination (min)^‡^	3.8 ± 0.21	3.6 ± 0.27	3.5 ± 0.24	3.3 ± 0.31	4.4 ± 0.3^α^	4.4 ± 0.41
d2 test						
TR Values^†^	279 ± 19	290 ± 20	288 ± 23	308 ± 23	246 ± 23	233 ± 32
TA Values^†^	97 ± 7.9	105 ± 9.5	97 ± 10	113 ± 11	96 ± 5.8	80 ± 16
O Values^‡^	23 ± 6.8	16 ± 5	27 ± 8.3	15 ± 6.5	10 ± 3.8	18 ± 3.4
C Values^‡^	11 ± 4.1	6.8 ± 2.8	13 ± 5	5.5 ± 3.3	4.7 ± 2.2	11 ± 5.1
O + C Values^‡^	35 ± 9.7	23 ± 8.1	40 ± 12	21 ± 11	15 ± 5.7	29 ± 8.2
TOT Values^†^	247 ± 18	268 ± 21	249 ± 22	287 ± 23	240 ± 25	204 ± 39
CON Values^†^	85 ± 11	99 ± 11	84 ± 14	108 ± 12	92 ± 4.1	70 ± 22
VAR Values^‡^	14 ± 2	12 ± 0.82	15 ± 2.3	12 ± 1.1	12 ± 4.5	12 ± 0.63
Oral SDMT-correct pairings^†^	25 ± 2.9	32 ± 2.6***	29 ± 3.3	35 ± 2.9**	17 ± 4.2	25 ± 4.8*
Oral SDMT-incorrect pairings^‡^	1.5 ± 0.36	1.1 ± 0.31	1.5 ± 0.31	1.1 ± 0.38	1.6 ± 1.1	1 ± 0.55
Oral SDMT-total pairings^†^	27 ± 2.9	34 ± 2.6**	30 ± 3.3	36 ± 2.7*	18 ± 4.2	26 ± 4.8
Digit span-forward^†^	6.8 ± 0.3	7.4 ± 0.56	7 ± 0.34	7.8 ± 0.62	6.2 ± 0.58	6.6 ± 1.2
Digit span-backward^†^	4.1 ± 0.43	4.9 ± 0.58	4.2 ± 0.59	4.9 ± 0.74	4 ± 0.32	4.8 ± 0.97
Digit span-total score^†^	11 ± 0.62	12 ± 1.1	11 ± 0.8	13 ± 1.3	10 ± 0.86	11 ± 2.2
Letter-number sequencing test^†^	5.4 ± 0.72	5.7 ± 0.82	5.7 ± 0.9	6.5 ± 0.96	4.6 ± 1.2	3.8 ± 1.4
Biochemical measurements						
Ammonia^‡^	39 ± 6.8	55 ± 9.8	40 ± 7.8	57 ± 13	38 ± 15	49 ± 13
IL6^‡^	3.7 ± 0.45	2.3 ± 0.24**	3.8 ± 0.54	2.2 ± 0.31**	3.7 ± 0.88	2.3 ± 0.41
IL18^†^	410 ± 39	268 ± 29**	450 ± 41	233 ± 29 ***	307 ± 80	359 ± 56
CCL20^‡^	80 ± 14	44 ± 6.3**	86 ± 15	41 ± 6.9**	66 ± 33	51 ± 15
CXCL13^†^	168 ± 15	145 ± 18	170 ± 18	123 ± 16**	164 ± 31	204 ± 41
IL22^‡^	70 ± 12	47 ± 7.5***	73 ± 15	48 ± 9.6**	63 ± 17	46 ± 12
IL15^†^	5.2 ± 0.48	3.2 ± 0.27**	5.6 ± 0.61	3.1 ± 0.36**	4.3 ± 0.64	3.4 ± 0.26
Fractalkine/CX3CL1^†^	728 ± 82	624 ± 75*	727 ± 73	663 ± 81	731 ± 249	523 ± 178

Values are expressed as mean ± SEM

PHES, Psychometric Hepatic Encephalopathy Score; DST, Digit Symbol Test; NCT-A, NCT-B: Number Connection Test A and B; SD, Serial Dotting Test; LTT, Line Tracing Test; TR, Total number of characters processed; TA, Total right answers; O, Total omission errors; C, Total commission errors; TOT, Total correctly processed; CON, Concentration performance; VAR, difference between maximum and minimum score. All biochemical parameters are in pg/mL, except ammonia levels, which are in µM. Stroop test: Congruent task: number of words read in 45 s; Neutral task: number of colours read in 45 s; Incongruent task: number of items completed in 45 s. Digit span and Letter-number sequencing: measured as number of right answers

^†^Parametric measurements. ^‡^ Non-parametric measurements. Differences between pre- and post- treatment were analysed using paired *T-test* for parametric measurements or paired Wilcoxon test for non-parametric measurements

Differences between responders and non-responders before treatment were analysed using: *T-test* if measurements were parametric or Wilcoxon test if measurements were not parametric

Resulting levels of significance were corrected using False Discovery Rate (FDR) method, and values of p < 0.05 after FDR correction were considered significant

Significant pre- and post-treatment differences are indicated by *: *p < 0.05, **p < 0.01, ***p < 0.001

Significant differences between response groups before treatment are indicated by α: ^α^p < 0.05

All observed improvements were greater when the analyses were restricted to responders, but besides the PHES subtests, no new tests showed previously unobserved changes. A similar effect was observed in all biochemical measurements except fractalkine. None of these changes were observed in non-responders, except for a significant improvement in the number of correct answers in oral SDMT (p < 0.05) (Table 2).

We found preexisting differences between responders and non-responders in the performance of both bimanual (p < 0.05) and visuomotor coordination tests (p < 0.05) (Table 2).

### Intra-network connectivity

**Fig. 3:**
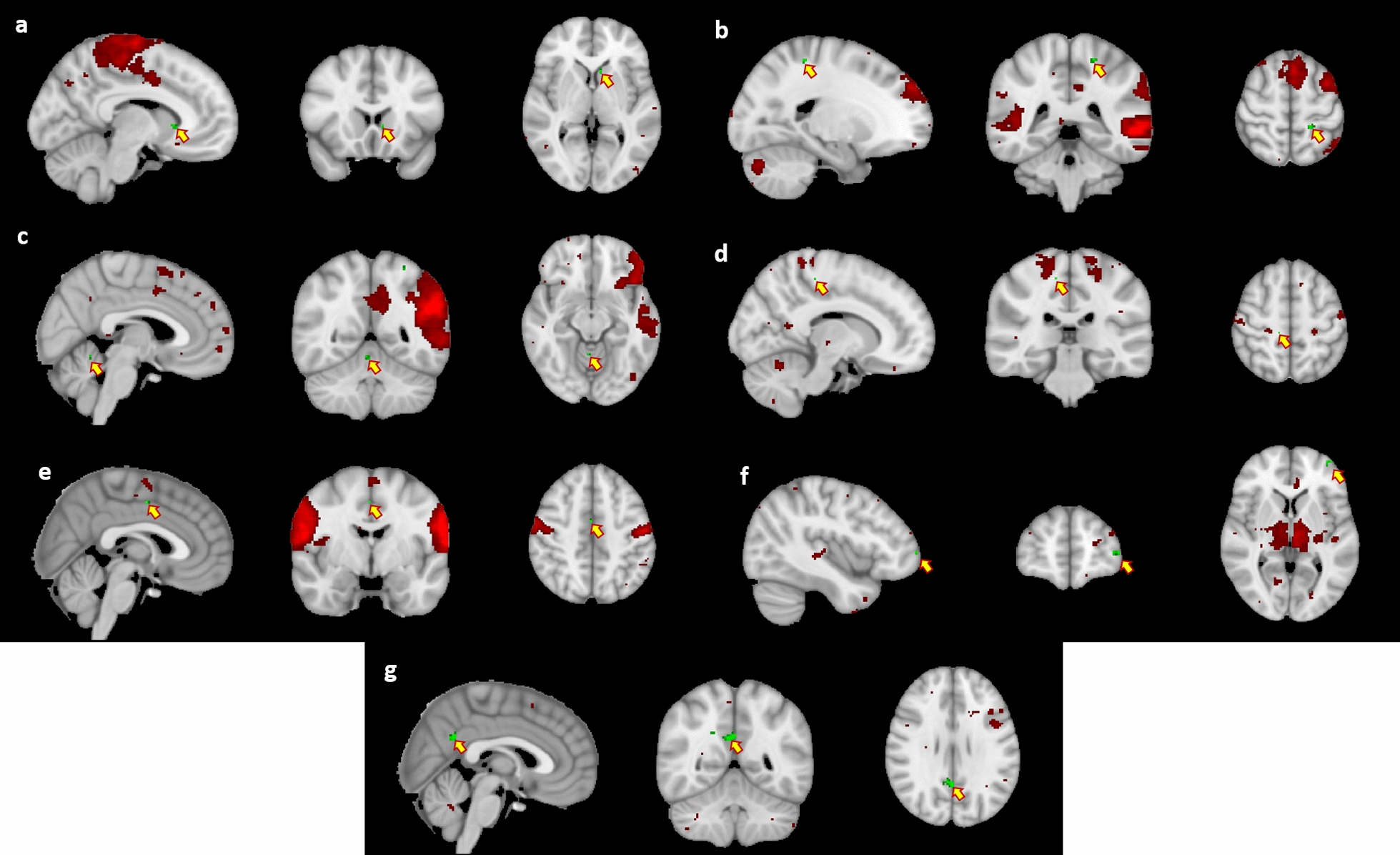
Spatial maps of all the clusters in which significant results were observed during the intra-network functional connectivity analysis. Clusters are shown as follows: **a** MSMN-1, **b** LFPN-1, **c** LFPN-2, **d** LSMN-1, **e** LSMN-2, **f** THN-1, **g** BGN-1. All clusters (green) are pointed (yellow arrows). The resting-state network (red) associated with each cluster is thresholded at 3 < Z < 10. All results were cluster-corrected for multiple comparisons using family-wise error (FWE), in combination with a threshold of p < 0.001 at the uncorrected voxel level. Clusters were considered significant at p < 0.05 after FWE correction. Images are shown following MNI convention

All changes and alterations described in this section, as well as their size and location, are included in Table 3. An increased connectivity after rifaximin treatment was observed in cluster MSMN-1 of the medial sensorimotor network (MSMN) in all patients, regardless of response group (Fig. 3A). A similar, non-significant tendency was observed in MSMN-2. On the other hand, reduced connectivity was observed in clusters LFPN-1 and LFPN-2 of the left fronto-parietal network (LFPN) (Fig. 3B and C), and in cluster LSMN-1 of the lateral sensorimotor network (LSMN) (Fig. 3D). Additional non-significant tendencies towards reduction in connectivity were also observed in clusters RFPN-1, LFPN-3 and LFPN-4, in both the right and left fronto-parietal networks (Table 3).

**Table 3:** Intra-network clusters showing significant changes and notable trends

Observed effect	Affected network	Cluster name	N voxels	p value	MNI (X, Y, Z)	Location
General effect of rifaximin						
Increase of signal after treatment	Medial sensorimotor network	MSMN-1	5	**0.01**	− 8, 18, 2	Left Caudate
Decrease of signal after treatment	Right fronto-parietal network	RFPN-1	1	0.096	− 30, − 62, 30	Left intraparietal 0 area (IP0)
	Left fronto-parietal network	LFPN-1	11	**0.02**	− 20, − 40, 58	Left Brodmann area 2
		LFPN-2	4	**0.034**	4,− 52,− 12	Right cerebellar lobule I-IV
		LFPN-3	2	0.051	− 28,− 52,62	Left area 7 anterior lateral (7AL)
		LFPN-4	1	0.094	26,− 2,30	Right superior corona radiata
	Lateral sensorimotor network	LSMN-1	1	**0.021**	12,− 30,54	Right corticospinal tract
Effect of treatment exclusively on responding patients						
Decrease of signal after treatment	Lateral sensorimotor network	LSMN-2	2	**0.01**	2,− 4,46	Right area 24 prime posterior (p24pr)
	Left fronto-parietal network	LFPN-5	1	0.074	18,10,16	Right caudate
		LFPN-6	1	0.099	26,− 2,28	Right superior corona radiata
Interaction between treatment and response group						
Relative increase of signal in Responder patients and Relative decrease of signal in Non-responder patients	Thalamic network	THN-1	10	**0.021**	− 41,57,4	Left area 9–46 anterior ventral (a9-46v)
		THN-2	2	0.086	− 3,− 33,12	Left Corpus callosum splenium
	Lateral visual network	LVN-1	1	0.089	− 26,− 38,52	Left Brodmann area 2
Pre-existing differences between response groups						
Increased signal in patients who will Respond	Basal ganglia network	BGN-1	39	**0.014**	2,− 54,28	Right area 7 medial (7 M)
		BGN-2	4	0.06	18,− 52,32	Right posterior corona radiata

The program FSL Randomise was used to analyse all intra-network differences

FSL Randomise uses Generalized Linear Models (GLM) to analyse differences between groups

All results were cluster-corrected for multiple comparisons using family-wise error (FWE), in combination with a threshold of p < 0.001 at the uncorrected voxel level

Clusters were considered significant at p < 0.05 after FWE correction

MNI: Montreal Neurological Institute; MSMN, medial sensorimotor network; RFPN, right fronto-parietal network; LFPN, left fronto-parietal network; LSMN, lateral sensorimotor network; THN, Thalamic network; LVN, lateral visual network; BGN, basal ganglia network

Significant p values are in bold

When restricting the sample to responding patients, decreased connectivity was observed in cluster LSMN-2 of the LSMN (Fig. 3E), as well as a similar non-significant tendency in clusters LFPN-5 and LFPN-6 of the LFPN, which was comparable to results from analysing the whole cohort.

An interaction between treatment effect and response group was observed in cluster THN-1 of the thalamic network (THN) (Fig. 3F). A non-significant trend was observed also in cluster THN-2, as well as in cluster LVN-1 of the lateral visual network (LVN). In all cases the observed interaction pointed to a relative increase of connectivity in responders and a relative decrease in non-responders (Table 3).

A pre-existing difference in connectivity between responders and non-responders was also found. Responders showed enhanced connectivity in cluster BGN-1, associated with their basal ganglia network (BGN) in comparison to non-responders (Fig. 3G). A similar, non-significant, tendency was observed in BGN-2.

The activity of networks whose activity was affected following rifaximin treatment (MSMN, LFPN and LSMN in all patients, and THN in responding patients) was compared with the activity of those networks in healthy controls. With the exception of LFPN, no significant differences were observed between patients after treatment and controls in any network. The differences observed in LFPN were roughly similar to the alterations detected by García-García et al. [15].

### Inter-network connectivity

**Fig. 4:**
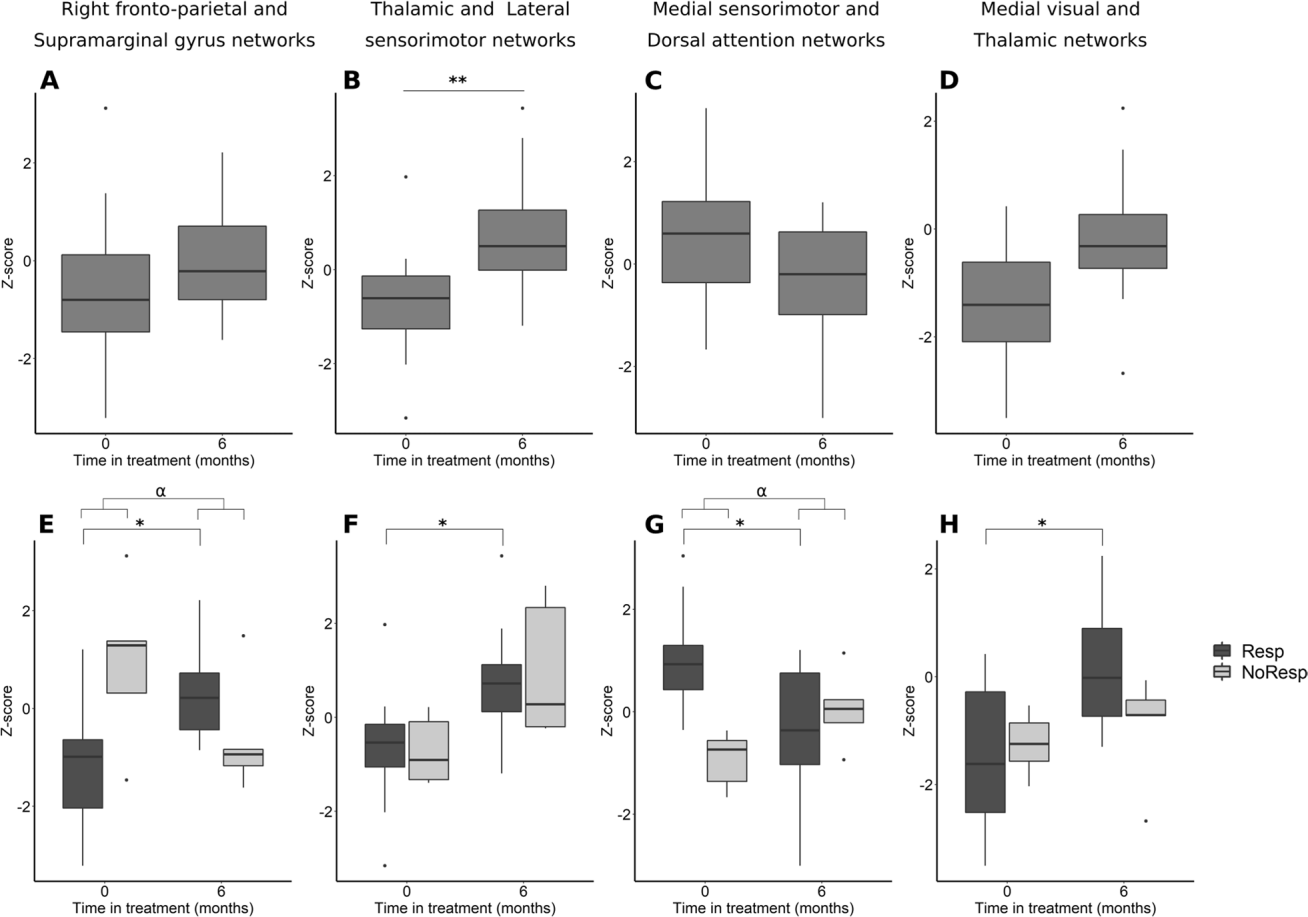
Boxplots showing z scores of inter-networks connections with significant changes after rifaximin treatment. **A**–**D** Considering all patients as one group, before and after treatment. **E**–**H** Stratifying patients by response group (Resp: responding patients; NoResp: non-responding patients). Significant pre- and post-treatment differences were analysed using a general linear model similar to a paired t-test and are indicated by (*): *p < 0.05, **p < 0.01. Significant interactions between treatment and response group were analysed using a general linear model similar to a 2-way mixed effect ANOVA and are indicated by (α): α p < 0.05. All p values were corrected for multiple comparisons using FDR, and differences were considered significant at p < 0.05 after correction

Our study of the effects of rifaximin independently of patient response revealed a significant change in THN-LSMN connectivity (Fig. 4B). The connection went from a slightly negative to a slightly positive value after treatment (p = 0.008). When considering each group separately, non-responders showed no significant change in this connection, but the change observed in responders remained significant (p = 0.027) (Fig. 4F).

The connection between the medial visual network (MVN) and THN evolved from a negative value to a more neutral one after treatment (Fig. 4D). However, this change was significant only when the analysis was restricted to responders (p = 0.034) (Fig. 4H), rather than all patients together (p = 0.053), although the latter trend nonetheless approached significance.

A significant interaction between treatment and response group was observed in the functional connectivity between the MSMN and the dorsal attention network (DAN) (p = 0.049) (Fig. 4G). In this case, responders showed an evolution from a positive towards a more neutral value, while non-responders connectivity progressed in the opposite direction. When considering the evolution of each group separately, only the change observed in responders remained significant (p = 0.026). Although the between-group differences in connectivity before the treatment were not significant (p = 0.063), they were nonetheless noteworthy (Fig. 4G).

Another significant interaction was observed in the connection between the right fronto-parietal network (RFPN) and the supramarginal gyrus network (SPGN) (p = 0.019) (Fig. 4E). In this case responders progressed from a negative value towards a more neutral one, while non-responders showed an evolution in the opposite direction (Fig. 4E). Along the same line as the above described connections, when analysing the response groups separately, non-responding patients showed no level of significance, but the changes experienced by responders remained significant (p = 0.026).

No connections between different networks showed significant or approaching significant differences when comparing the initial state of the two response groups.

### Correlation analysis

Results of correlations between intra-network changes and psychometric tests showed that PHES score, DST and Oral SDMT correlated with both clusters related to the fronto-parietal network. DST and Oral SDMT showed a high correlation with LSMN-1 as well (Table 4). Clusters MSMN-1, LSMN-2, THN-1 and BGN-1 showed no significant correlations with any psychometric tests.

**Table 4:** Significant correlations between remarkable intra-network functional clusters and neuropsychological tests or blood biochemical parameters

Network	Cluster	Psychometric tests	R	p value
Left fronto-parietal network	LFPN-1	PHES	− 0.595	0.046
		DST (items completed)	− 0.583	0.046
		NCT-A (seconds)	0.708	0.015
		Oral SDMT (correct pairings)	− 0.668	0.019
		Oral SDMT (total pairings)	− 0.6	0.019
	LFPN-2	PHES	− 0.7	0.015
		DST (items completed)	− 0.683	0.019
		LTT (seconds + errors)	0.597	0.029
		Stroop-neutral task	− 0.644	0.02
		Stroop-incongruent task	− 0.592	0.034
		Oral SDMT (correct pairings)	− 0.769	0.013
		Oral SDMT (total pairings)	− 0.704	0.015
Lateral sensorimotor network	LSMN-1	DST (items completed)	− 0.69	0.018
		Oral SDMT (correct pairings)	− 0.669	0.018

Network	Cluster	Biochemical parameter	R	p value
Left fronto-parietal network	LFPN-1	IL6	0.66	0.019
	LFPN-2	IL6	0.815	< 0.001
		IL18	0.565	0.026
		Mip3/CCL20	0.59	0.024
		IL22	0.656	0.01
Lateral sensorimotor network	LSMN-1	IL6	0.663	0.009
		IL18	0.524	0.046
		IL22	0.755	0.002
		IL15	0.511	0.046
		Fractalkine/CX3CL1	0.519	0.046
	LSMN-2	IL6	0.68	0.017
		IL18	0.824	< 0.001
		Mip3/CCL20	0.711	0.017
		CXCL13	0.66	0.018
		IL22	0.607	0.032
		IL15	0.689	0.017
Medial sensorimotor network	MSMN-1	Ammonia	0.55	0.03
		IL6	− 0.709	0.003
		IL22	− 0.715	0.003
		IL15	− 0.597	0.021

R and p from significant Spearman correlations are shown

R, correlation coefficient. Correlations were considered significant at p < 0.05 after FDR correction

Clusters are named as shown in Table 3

Observing the correlations between intra-network alterations and biochemical parameters, all clusters related to the general effect of rifaximin were significantly correlated with at least one inflammatory parameter (Table 4). Ammonia levels showed a significant correlation with intra-network connectivity only in the MSMN-1 cluster. The presence of IL6 was particularly notable, as it was the only biochemical measurement that correlated with all the included clusters. IL15, IL18 and IL22 had a remarkable presence as well, with LFPN-1 being the only cluster that did not correlate with at least one of them. All significant correlations of clusters LFPN-1, LFPN-2, LSMN-1 and LSMN-2 with all biochemical parameters were positive, while those of MSMN-1 were negative.

When we analysed the THN-1 correlations in the total group of treated MHE patients, no significant correlations were observed. Given that the evolution of this cluster is opposite in responder and non-responder patients (Table 3), we performed the correlations in the responder group to assess whether the change in THN-1 signal was accompanied by improvements in cognitive or biochemical parameters. As for the correlations with the psychometric tests, only the incongruent Stroop task and the SDMT scores tended to be significant (r = 0.5, p = 0.06 and r = 0.49, p = 0.07, respectively) but the small sample size and the correction of the p-values made them less significant. For biochemical parameters, significant correlations were observed with IL18 (r = − 0.61; p = 0.008) and IL15 (r = − 0.63; p = 0.007), which after correction for p-values became trends (p = 0.07 and p = 0.06, respectively) (Additional file 1: Table S2).

There were no significant correlations between functional connectivity in BGN-1 cluster and psychometric or biochemical parameters.

Several significant results also emerged from analysis of correlations between inter-network connections and psychometric tests (Table 5). In this case, however, the only connection that showed significant correlations was the MVN-THN connection. This connection correlated significantly with several scores in almost all psychometric tests performed, including tests assessing cognitive flexibility (Stroop test), mental processing speed (Oral SDMT, and DST from the PHES battery) and sustained concentration (d2 test). Subtest LTT from the PHES battery and the Bimanual coordination test, which evaluate motor coordination, were significantly correlated with the MVN-THN connection as well (Table 5).

**Table 5:** Significant correlations between remarkable inter-network functional connections and neuropsychological tests or blood biochemical parameters

Connection	Psychometric tests	R	p value
Medial visual network and Thalamic network	PHES	0.723	0.01
	DST (items completed)	0.788	0.006
	NCT-B (seconds)	− 0.778	0.008
	LTT (seconds + errors)	− 0.57	0.047
	Stroop-neutral task	0.666	0.022
	Stroop-incongruent task	0.669	0.022
	Bimanual coordination (min)	− 0.892	0.002
	d2 test-TR values	0.627	0.047
	d2 test-TA values	0.638	0.046
	d2 test-TOT values	0.681	0.03
	d2 test-CON values	0.62	0.047
	Oral SDMT (correct pairings)	0.753	0.009
	Oral SDMT (total pairings)	0.731	0.01

Connection	Biochemical parameters	R	p value
Medial sensorimotor network and Dorsal attention network	IL18	0.581	0.041
	CXCL13	0.633	0.033
Right fronto-parietal network and Supramarginal gyrus network	IL6	− 0.633	0.035
	IL18	− 0.774	< 0.001
	IL15	− 0.59	0.043

R and p from significant Spearman correlations are shown. R, correlation coefficient. Correlations were considered significant at p < 0.05 after FDR correction

Significant correlations between inter-network connections and biochemical measurements were also observed, but quite limited. IL18 correlated significantly with both the MSMN-DAN and RFPN-SPGN connections (Table 5). CXCL13 showed a significant correlation with the MSMN-DAN connection as well, while IL6 and IL15 correlated with the RFPN-SPGN connection.

## Discussion

In this study, we assessed the effects of rifaximin on the activity of different resting-state networks in MHE patients, and on the functional connectivity between them. Besides this, we also investigated pre-existing differences between patients who did or did not respond to treatment. Finally, we analysed the relationship between these alterations and different psychometric and biochemical alterations also observed in patients with MHE.

It is important to note that, given the non-absorbable nature of rifaximin, all the effects observed in this study would be an indirect result of its effects on the gut microbiota and the regulation of inflammatory imbalances present in patients with cirrhosis [37, 42].

### Effect on sensorimotor, fronto-parietal, and thalamic networks following rifaximin treatment

Most intra-network effects were observed after rifaximin treatment on a general level, encompassing all treated patients. We observed generally increased sensorimotor network activity in the left caudate, and generally decreased left fronto-parietal network activity in the left Brodmann area 2. Both these abovementioned effects after rifaximin treatment have previously been reported in MHE patients performing N-back and inhibitory control tests with fMRI [58]. Additionally, thalamic network activity in area a9-46v was relatively increased in responders compared to non-responders. Area a9-46v is part of the dorsolateral prefrontal cortex (DLPFC), which is involved in executive function. The effects observed in the thalamic network function after rifaximin treatment could be related to improved thalamus function and structure, which have been reported as deteriorated in MHE patients [23, 59]. A significant reduction of the connectivity of the lateral sensorimotor network was observed as well, but the clusters we found were very small, and of dubious biological significance.

Studying inter-network connections, a general effect was observed after rifaximin treatment in the connection between the thalamic and lateral sensorimotor networks, which went from a negative to a positive z-score. Other inter-network connections also underwent significant changes, but in these cases among responding patients only. These included connections between medial sensorimotor and dorsal attention network (involved in visuospatial attention) the right fronto-parietal and supramarginal gyrus network (somatosensory perception) and the thalamic and medial visual network (visuospatial perception).

Taken together, these results suggest that rifaximin indirectly helps improve communication between brain networks and areas mainly involved in executive function (i.e.: the fronto-parietal and dorsal attention networks, and the DLPFC), in areas that play a role mainly in processing different stimuli (i.e.: the thalamus, Brodmann area 2, supramarginal gyrus and visual network), which provide the sensorial information necessary for executive functions to be performed. Although seemingly less remarkable, changes in communication with areas involved in motor control (i.e.: sensorimotor network and caudate) were also observed.

Several significant results were observed upon performing correlation analyses between the above changes and patient cognitive performance. Most tests were present to a certain level in these analyses, but the most prevalent were the Oral-SDMT and other tests that evaluate functions such as attention, and mental processing speed. Most of the significant correlations we observed involved clusters related to the activity of the left fronto-parietal network, which is directly involved in these and other executive functions.

The correlation between the cognitive performance of the patients and the connection between the medial visual and the thalamic network was remarkable as well. This could be due to the fact that, even though this connection evolved in a similar direction both in responding and non-responding patients, the changes observed were significant only when limited to the responding patients; a similar pattern to that observed in most cognitive tests. These correlations would suggest a relationship between the improvement of the visuospatial perception and processing of the patients, and their cognitive performance, especially in attention related tests.

### Reduction of aberrant hyperconnectivity following rifaximin treatment

In most cases, the connections between different networks seemed to evolve towards greater independence from each other after treatment. Similarly, global intra-network changes in connectivity of the fronto-parietal and lateral sensorimotor networks followed a similar tendency of decreased connectivity after treatment. These results suggest that the treatment improves brain function by ameliorating aberrant hyperconnectivity suffered by MHE patients. This pattern has already been observed in MHE and in other pathologies, such as multiple sclerosis and traumatic brain injury [19, 21, 60].

### Rifaximin treatment does not recover a normal brain function

Of the networks affected by rifaximin treatment, only the left fronto-parietal network has been previously identified as altered in MHE patients compared to healthy controls [15]. The activity of the sensorimotor and thalamic networks, which were shown to be affected by rifaximin in this study, has not shown significant alterations in previous studies, so it was expected to neither observe significant differences between patients after treatment and controls when studying the activity of these networks.

Despite the effects of rifaximin, the left fronto-parietal network remained similarly altered. These and other alterations in connectivity were largely still present in patients after treatment. This result is not unexpected, considering that the changes driven by rifaximin in left fronto-parietal network connectivity occurred at different locations from those caused by MHE, and that they further decreased connectivity, rather than increasing it to a state approaching that of a healthy individual [15]. Besides this network, there seem to be no other common point between the networks altered in patients with MHE and those affected by rifaximin. Altogether, these results suggest that 6 months of rifaximin treatment improves cognitive function at a neurological level in responding patients yet the mechanisms involved do not necessarily include reversal of MHE-related alterations, thus pointing to a certain level of redundancy in the neural circuits involved in the process [61]. On the other hand, the prevalence of the alterations caused by MHE suggest a high risk of relapse if patients abandon treatment [62].

### Reduction of inflammation, but not ammonia levels after rifaximin treatment

Comparing inflammatory cytokine levels before and after treatment, most decreased significantly in responders, but not in non-responders. Even taking a lack of significance due to the small patient sample size into account, IL6 and IL22 were the only cytokines that evolved in a similar way regardless of group response. In other cases, non-responders showed lesser reduction in levels, or directly increased levels, as observed with IL18 and CXCL13. Most of these cytokines show significant correlations with several significant changes revealed in brain function analysis. This points to the already observed effect of inflammation on the brain function in MHE patients, and how its decrease, promoted by rifaximin, helps restore these functions. Similar results have previously been reported, particularly regarding CCL20, CX3CL1, and IL15 levels, which are known to promote lymphocyte infiltration into the brain [42]. Anti-inflammatory effects of rifaximin could be mediated by induction of the expression of pregnane-X-receptor (PXR) in intestinal epithelial cells [63], promoting the transcription of genes for detoxification enzymes and cytokines, ultimately reducing inflammation and improving MHE.

In contrast, no significant decrease in ammonia levels was observed in any patient group after treatment. Lower ammonia levels were reported in patients with overt HE grade I or II after rifaximin treatment [64], but ammonia reduction with rifaximin was not statistically significant in MHE patients [35]. It should be noted, however, that the blood ammonia levels of our study patients were not as high as those of patients with overt HE, which could explain why rifaximin did not alter them substantially.

These results suggest that the improved inflammatory state of MHE patients, and the resulting reduction of lymphocyte infiltration, following rifaximin treatment is enough to favour the observed changes in brain function and enhanced cognitive performance.

### Responding and non-responding patients show pre-existing differences

When considering intra-network connectivity, a difference was observed in the connectivity of the basal ganglia network. It was located in the right medial part of the Brodmann area 7, which is part of the precuneus, and was the largest cluster found in this study. Both the precuneus and basal ganglia play important roles in executive functions such as working memory and visuospatial attention, which have already been observed in this study to improve after treatment in responders. Alterations located in the precuneus and/or related to a decreased connectivity of the basal ganglia have been already reported in patients with MHE [15, 16]. These results indicate that patients responding positively to rifaximin have less deteriorated connectivity in this specific area than patients who do not respond to this treatment.

The lack of significant correlation between pre-existing differences in basal ganglia activity and any neuropsychological test or biochemical parameter would suggest that this difference in connectivity is not related to a remarkable difference in inflammation, hyperammonemia or cognitive performance between groups before treatment.

The main limitation of this study is the small study sample (subdivided even further in certain analyses), which particularly affected the non-responder group and reduced the statistical power of the results obtained. However, the conditions for results to be statistically significant were very strict, because we performed systematic corrections of p-values. Additionally, due to the limitation in sample size and computational power, it was not possible to include all the psychometric and inflammatory measurements as regressors in the general linear models of the performed analyses. In most cases, however, the changes observed in these variables were consistent with the separation of patients in responding and non-responding groups.

Another limitation could be the lack of a placebo group and the open-label design. However, results of this study could be the basis of future randomised, double blind and placebo controlled clinical trials. This study is an exploratory study for characterizing brain functional connectivity modulation by rifaximin treatment that could be useful for future, placebo-controlled trials in MHE utilizing brain MR imaging.

## Conclusions

In conclusion, although rifaximin does not directly correct most functional alterations caused by MHE, it favours subtle changes in brain function which frequently correct aberrant hyperconnectivity, and which are related to improvement in different executive functions as well as pro-inflammatory cytokine normalization in patients who respond favourably to treatment. This improvement, however, does not extend to blood ammonia levels in MHE patients. We also found pre-existing increased connectivity in the precuneus of patients who showed a favourable response to treatment. Finally, the results obtained in this study using the analysis of inter-network connections via FSLNets and graph theory show the potential of an approach rarely applied in the study of functional connectivity, and completely novel in the study of rifaximin effects on MHE.

### Supplementary Information

**Additional file 1: Table S1.** Psychometric and biochemical characteristics of controls, nMHE patients and MHE patients. **Table S2.** Correlations between changes in THN-1 cluster and psychometric and biochemical parameters in the group of patients responding to rifaximin treatment.

## Data Availability

Data is contained within the article and Additional files.

## Abbreviations

BGN: Basal ganglia network
DAN: Dorsal attention network
DLPFC: Dorsolateral prefrontal cortex
DST: Digit Symbol Test
fMRI: Functional magnetic resonance imaging
HE: Hepatic encephalopathy
ICA: Independent component analysis
LFPN: Left fronto-parietal network
LSMN: Lateral sensorimotor network
LVN: Lateral visual network
MELODIC: Multivariate Exploratory Linear Optimized Decomposition into Independent Components
MHE: Minimal hepatic encephalopathy
MSMN: Medial sensorimotor network
MVN: Medial visual network
nMHE: Patients without minimal hepatic encephalopathy
Oral SDMT: Symbol Digit Modalities test, oral version
PHES: Psychometric Hepatic Encephalopathy Score
RFPN: Right fronto-parietal network
SPGN: Supramarginal gyrus network
THN: Thalamic network
