# Supplementary material for: Improved cognition after rifaximin treatment is associated with changes in intra- and inter-brain network functional connectivity
Source: J Transl Med. 2024 Jan 12;22:49. doi: 10.1186/s12967-023-04844-7 (PMC10787503; doi:10.1186/s12967-023-04844-7)
Supplement: Supplementary file 1 — Additional file 1: Table S1. Psychometric and biochemical characteristics of controls, nMHE patients and MHE patients. Table S2. Correlations between changes in THN-1 cluster and psychometric and biochemical parameters in the group of patients responding to rifaximin treatment. [file 12967_2023_4844_MOESM1_ESM.docx]

**SUPPLEMENTARY TABLES**

**Improved cognition after rifaximin treatment is associated with changes in intra- and inter-brain network functional connectivity**

Franc Casanova, Juan-José Gallego, Alessandra Fiorillo, Amparo Urios, María-Pilar Ríos, José Luis León, María-Pilar Ballester, Desamparados Escudero-García, Elena Kosenko, Vicente Belloch, Carmina Montoliu^*^

**Contents:**

**Table S1.** Psychometric and biochemical characteristics of controls, nMHE patients and MHE patients.

**Table S2.** Correlations between changes in THN-1 cluster and psychometric and biochemical parameters in the group of patients responding to rifaximin treatment.

**Table S1.** Psychometric and biochemical characteristics of controls, nMHE patients and MHE patients.

| Neuropsychological tests | Controls | nMHE patients | | MHE patients |
| --- | --- | --- | --- | --- |
| PHES Global score ^§^ | 0.95 ± 0.25 | -1 ± 0.27** | | -7.3 ± 0.85*** ^ααα^ |
| DST (items completed) ^†^ | 44 ± 2.2 | 26 ± 1.7*** | | 21 ± 2*** ^α^ |
| NCT-A (seconds) ^§^ | 29 ± 1.6 | 41 ± 3.3** | | 74 ± 11*** ^α^ |
| NCT-B (seconds) ^§^ | 73 ± 5.8 | 116 ± 9.4** | | 228 ± 39*** ^αα^ |
| SD (seconds) ^§^ | 60 ± 2.7 | 80 ± 3.7** | | 119 ± 8.3*** ^ααα^ |
| LTT (seconds + errors) ^§^ | 98 ± 3.5 | 125 ± 6.7** | | 210 ± 14*** ^ααα^ |
| Stroop-congruent task ^†^ | 119 ± 2.8 | 98 ± 3*** | | 76 ± 4.2*** ^ααα^ |
| Stroop-neutral task ^†^ | 83 ± 3.1 | 74 ± 3 | | 56 ± 2.3*** ^ααα^ |
| Stroop-incongruent task ^†^ | 47 ± 2 | 39 ± 2.3* | | 28 ± 2.1*** ^αα^ |
| Bimanual coordination (min) ^§^ | 1.9 ± 0.04 | 2.2 ± 0.05** | | 3.3 ± 0.37*** ^αα^ |
| Visuo-motor coordination (min) ^‡^ | 2.4 ± 0.08 | 2.8 ± 0.11** | | 3.8 ± 0.21*** ^αα^ |
| d2 test |  |  | |  |
| TR Values ^†^ | 428 ± 16 | 295 ± 19*** | | 279 ± 19*** |
| TA Values ^†^ | 159 ± 7.6 | 104 ± 7.3*** | | 97 ± 7.9*** |
| O Values ^§^ | 23 ± 5.6 | 21 ± 7.5 | | 23 ± 6.8 |
| C Values ^§^ | 1 ± 0.31 | 6.8 ± 3.5** | | 11 ± 4.1** |
| O+C Values ^§^ | 24 ± 5.7 | 28 ± 8.3 | | 35 ± 9.7 |
| TOT Values ^†^ | 404 ± 16 | 271 ± 17*** | | 247 ± 18*** |
| CON Values ^†^ | 158 ± 7.8 | 97 ± 8*** | | 85 ± 11*** |
| VAR Values ^§^ | 14 ± 0.82 | 13 ± 1.6 | | 14 ± 2 |
| Oral SDMT-correct pairings ^‡^ | 51 ± 1.3 | 36 ± 2.4*** | | 25 ± 2.9*** ^α^ |
| Oral SDMT-incorrect pairings ^§^ | 1.1 ± 0.35 | 1.9 ± 0.52 | | 1.5 ± 0.36 |
| Oral SDMT-total pairings ^‡^ | 52 ± 1.1 | 38 ± 2.1*** | | 27 ± 2.9*** ^αα^ |
| Digit span-forward ^§^ | 9.5 ± 0.48 | 7.5 ± 0.28** | | 6.8 ± 0.3*** |
| Digit span-backward ^§^ | 6.7 ± 0.61 | 4.6 ± 0.28** | | 4.1 ± 0.43*** |
| Digit span-total score ^§^ | 16 ± 0.98 | 12 ± 0.48** | | 11 ± 0.62*** |
| Letter-number sequencing test ^†^ | 10 ± 0.54 | 6.8 ± 0.48** | | 5.4 ± 0.72*** |
| Biochemical determinations | | |  |  |
| Ammonia ^§^ | 9.1 ± 0.72 | 22 ± 5.2 * | | 39 ± 6.8 ***  ^αα^ |
| IL6 ^§^ | 1.5 ± 0.07 | 2.6 ± 0.36** | | 3.7 ± 0.45*** ^α^ |
| IL18 ^‡^ | 153 ± 18 | 240 ± 26* | | 410 ± 39*** ^αα^ |
| Mip3/CCL20 ^§^ | 9.7 ± 1.2 | 54 ± 7.5*** | | 80 ± 14*** |
| CXCL13 ^‡^ | 57 ± 2.7 | 124 ± 9*** | | 168 ± 15*** ^α^ |
| IL22 ^§^ | 40 ± 4.5 | 51 ± 3.5 | | 70 ± 12* |
| IL15 ^§^ | 2.9 ± 0.22 | 6.1 ± 0.77*** | | 5.2 ± 0.48*** |
| Fractalkine/CX3CL1 ^§^ | 419 ± 21 | 597 ± 46** | | 728 ± 82*** |

Values are expressed as mean ± SEM. MHE, minimal hepatic encephalopathy. nMHE, patients without MHE. PHES, Psychometric Hepatic Encephalopathy Score; DST, Digit Symbol Test; NCT-A, NCT-B: Number Connection Test A and B; SD, Serial Dotting Test; LTT, Line Tracing Test; d2 test: TR, Total number of characters processed; TA, Total right answers; O, Total omission errors; C, Total comission errors; TOT, Total correctly processed; CON, Concentration performance; VAR, difference between maximum and minimum score. All biochemical parameters are in pg/mL, except ammonia levels, which are in µM. Stroop test: Congruent task: number of words read in 45 seconds; Neutral task: number of colours read in 45 seconds; Incongruent task: number of items completed in 45 seconds. Digit span and Letter-number sequencing tests: measured as number of right answers. When comparing controls, nMHE patients and MHE patients, differences between groups were analyzed using (^†^) one-way ANOVA followed by post-hoc Tukey for parametric and homoscedastic measurements, (^‡^) Welch's ANOVA followed by post-hoc Games-Howell test if measurements were parametric but not homoscedastic, or (^§^) Kruskal-wallis followed by post-hoc Dunn test for non-parametric measurements. Resulting levels of significance were corrected using False Discovery Rate method. Differences were considered significant at p<0.05 after False Discovery Rate correction. Significant differences of MHE and nMHE patients compared to controls are indicated by *: * p<0.05, ** p<0.01, *** p<0.001. Significant differences of MHE patients compared to nMHE patients are indicated by α: α p<0.05, αα p<0.01, ααα p<0.001

**Table S2**. Correlations between changes in THN-1 cluster from thalamic network and psychometric and biochemical parameters in the group of patients responding to rifaximin treatment.

| **Psychometric tests** | **R** | **Uncorrected p value** | **corrected p value** |
| --- | --- | --- | --- |
| Stroop-incongruent task | 0.503 | 0.067 | 0.579 |
| Oral SDMT (correct pairings) | 0.494 | 0.073 | 0579 |
| **Biochemical parameters** | **R** |  | **p value** |
| IL18 | -0.611 | 0.008 | 0.071 |
| IL15 | -0.631 | 0.007 | 0.066 |

R and p from significant Spearman correlations are shown. R, correlation coefficient. Correlations were considered significant at p<0.05 after FDR correction.
